# Risk factor management over two decades in hospitalised patients with chronic limb-threatening ischaemia with and without diabetes mellitus

**DOI:** 10.1177/20420188251362729

**Published:** 2025-08-22

**Authors:** Sofia Bodinger, Tove Wikström, Anders Gottsäter, Stefan Acosta

**Affiliations:** Department of Clinical Sciences, Lund University, Malmö, Sweden; Department of Clinical Sciences, Lund University, Malmö, Sweden; Department of Clinical Sciences, Lund University, Malmö, Sweden; Department of Medicine, Skåne University Hospital, Malmö, Sweden; Department of Clinical Sciences, Lund University, Ruth Lundskogsg 10, Malmö 205 02, Sweden; Vascular Centre, Department of Cardiothoracic and Vascular Surgery, Skåne University Hospital, Malmö, Sweden

**Keywords:** chronic limb-threatening ischaemia, diabetes mellitus, hospitalisation, incidence, management, prognosis

## Abstract

**Background:**

Chronic limb-threatening ischaemia (CLTI) causes high rates of amputation and mortality.

**Objectives:**

To compare incidence, management and prognosis in hospitalised patients with CLTI with and without diabetes mellitus (DM) in 2001 and 2023. A secondary objective was to compare adherence to global vascular guidelines on risk factors between patients with and without DM in 2023.

**Design:**

Retrospective study.

**Methods:**

Group differences were tested using the Mann–Whitney *U* test, independent sample *t* test or the Chi-square test, as appropriate. The effects of DM on major amputation or mortality at 1 year were evaluated in a multivariable logistic regression model according to a directed acyclic graph.

**Results:**

The incidence of hospitalisations for CLTI was reduced from 37.4 (95% confidence interval (CI), 33.3–41.6) in 2001 to 22.8 (95% CI, 19.7–25.8) per 100,000 person-years in 2023. The proportion of patients on full-dose oral anticoagulant therapy (*p* < 0.001) and lipid-lowering treatment (*p* < 0.001) increased significantly between the two time periods. In 2023, Wounds, Ischemia and foot Infection-classification in all patients with foot ulcers was documented in 6.9%. Anaemia was present at hospital admission in 67.0% and 52.5% of patients with CLTI with and without DM, respectively (*p* = 0.031). Endovascular therapy was performed more often in those with DM compared to those without DM (*p* = 0.004). Antiplatelet therapy (*p* = 0.008) and smoking cessation interventions (*p* = 0.033) were offered less often to those with DM. DM (odds ratio (OR), 1.7 (95% CI, 1.02–2.83)) was independently associated with increased mortality at 1 year, whereas period 2023 as opposed to 2001 (OR, 0.62 (95% CI, 0.38–0.99)) was associated with decreased mortality.

**Conclusion:**

The incidence of hospitalisation for CLTI appears to have been reduced, and medical care of patients with CLTI has improved prognosis. Nevertheless, there is still room for large improvements of secondary prevention care in patients with CLTI, particularly in those with DM.

## Plain language summary

In-hospital management over two decades in patients with advanced peripheral vascular disease with or without diabetes

The present study evaluates the frequency of hospitalization, management of risk factors such as cholesterol levels and smoking, and prognosis in 2001 compared to 2023 in patients with advanced peripheral vascular disease with or without diabetes mellitus in a vascular surgery unit in Sweden. In addition, adherence to management of risk factors according to global vascular guidelines published in 2019 were evaluated in the 2023 patient cohort. The frequency of hospitalized patients decreased, the patients on blood thinner medication and cholesterol lowering therapy increased. Low levels of hemoglobin responsible for oxygen supply was common in both patients with and without diabetes. Medication against arterial calcification and advice against stop smoking was offered less to patients with Diabetes. Diabetes was a risk factor for death at 1 year, whereas patients survived better in 2023 than 2001. Medical care has improved in these patients, but there is still room for improvement, particularly in those with diabetes.

## Introduction

The end stage of peripheral artery disease (PAD) is chronic limb-threatening ischaemia (CLTI).^1^ The diagnosis of CLTI is based on previously documented PAD caused by atherosclerosis, and rest pain (associated with ischaemia) or tissue loss (from ulceration or gangrene). To be classified as CLTI, the symptoms must persist for 2 weeks or more and be confirmed with appropriate hemodynamic measurements. If the diagnosis of CLTI is based on rest pain, it should be associated with abnormal values of the hemodynamic measurements, and if the diagnosis is based on tissue loss it should be graded using a prognostic classification system such as Wounds, Ischaemia and foot Infection (WIfI).^1,2^ The likelihood of amputation after 1 year in patients with CLTI increases with higher WIfI stages.^2^ The first course of action is antithrombotic-, lipid-lowering- and antihypertensive medication, optimisation of glucose control in patients with Diabetes Mellitus (DM), and management of pain.^1^ The possibility for surgery depends on the evaluation of risk factors, location and extent of arterial diseases. The different surgical options are endovascular therapy, open vascular surgery and amputation. In some patients, conservative treatment with medical therapy may be preferred. The end stage treatment is palliative care. CLTI is linked to decreased quality of life and high annual amputation and mortality rates of 14.8% and 20.5%, respectively.^3^

The Global Vascular Guidelines (GVG) on the Management of CLTI^1^ can be used to improve evidence-based treatments and call attention to research needs. The GVG recommends different treatments for patients with CLTI, like platelet aggregation inhibitors or the regime used in the Voyager PAD study^4^ (2.5 mg rivaroxaban twice and 75 mg aspirin once daily) to reduce the risk of ischaemia, thrombosis and embolisation. The guidelines also recommend moderate or high-intensity statins to reduce the risk of CVD by lowering LDL cholesterol and stabilising atherosclerotic plaques.^1,5^ Another recommendation in the guideline from 2019 was that the primary glucose lowering agent for patients with type 2 DM should be metformin.^1^

Other recommendations from the GVG include frequent measurement of blood pressure (BP) during hospitalisation, the use of the WIfI classification system, as well as assessment of neuropathy and hemodynamic measurements for limb staging and diagnosis.

The guidelines also offer recommendations on lifestyle modifications. These include asking patients about smoking and offering smoking cessation intervention if necessary, giving advice on reducing the intake of saturated fats and carbohydrates, and increasing the intake of monounsaturated fats, omega-3, antioxidants, vegetables and fruits. Recommendations about physical activity are expressed as both regular exercise and walking-based exercise after surgery. The World Health Organisation (WHO) recommends 150–300 min of moderate intensity exercise per week for adults.^6^

The primary aim of this study was to compare incidence, risk factors, treatment and outcomes in hospitalised patients with CLTI with and without DM in 2023 and 2001.^7^ The secondary aim was to compare differences of risk factor management according to the GVG on the management of CLTI^1^ between those with and without DM during 2023.

## Methods

### Study setting

This retrospective study compares characteristics of CLTI patients in 2001^7^ and 2023. The Department of Vascular Diseases at Skåne University Hospital, Malmö, is responsible for vascular surgical care in southern Sweden. Patients were identified with the International Classification of Diseases diagnosis code I702, I702A, I702C or I702D. Patients with CLTI and DM were hospitalised at the Department of Vascular Diseases in 2001, and at both the Department of Vascular Diseases and Endocrinology in 2023 prior to lower extremity revascularisation.

### Selection of subjects

#### Inclusion criteria

Patients diagnosed and hospitalised with CLTI in 2001 and 2023.

#### Exclusion criteria

Patients <18 years of age and patients with claudication.

### Definitions

CLTI was defined as PAD with rest pain or foot ulcer for more than 2 weeks.^8^

Hypertension was defined as antihypertensive treatment, a diagnosis of hypertension, systolic BP >140 mmHg or diastolic BP >90 mmHg.^9^ Antiplatelet treatment was defined as aspirin or clopidogrel. Oral anticoagulant treatment was defined as warfarin or direct acting oral anticoagulants. Advice on diet was defined as recommendations of less carbohydrates or saturated fats or more fruits and vegetables.

Obesity was defined as body mass index (BMI) >30 kg/m^2^. Advice against obesity was defined as recommendation of physical activity, dietary changes and/or weight-reducing medication.

Moderate and high-intensity statins were defined as 40 or 80 mg of any statin or 20 or 40 mg of rosuvastatin at discharge. Lipid-lowering treatment was defined as low, moderate or high dose of any statin or other lipid-lowering medication. Regime used in the Voyager PAD study was defined as rivaroxaban 2.5 mg twice and aspirin 75 mg daily.^4^ Ischaemic heart disease (IHD) was defined as coronary artery disease or previous myocardial infarction. Cerebral vascular accident (CVA) was defined as previous cerebral infarction or spontaneous nontraumatic cerebral bleeding.

Concomitant atherosclerotic diseases were defined as IHD, CVA or both. DM was defined as fasting blood glucose ⩾7.0 mmol/L twice, accidental blood glucose ⩾11.0 mmol/L twice or a previous diagnosis of DM. Anaemia was defined as haemoglobin <134 g/L for men and <117 g/L for women. Glomerular filtration rate (GFR) was estimated by available data on age, sex, creatinine, weight and height by using an eGFR calculator.

### Statistical methods

**Figure 1.**
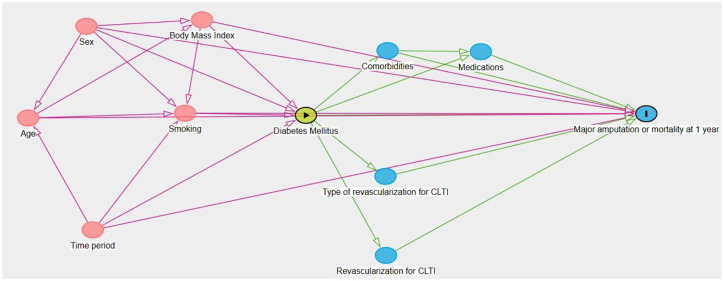
Minimum sufficient adjustment sets for estimating the total effect of DM on major amputation/mortality at 1 year in patients with DM and CLTI. Directed acyclic graph over potential interactions between variables. DM is the exposure and major amputation or mortality at 1 year are the outcomes. Variables in pink could be potential confounders. Variables in blue are mediators. The multivariable analysis evaluating the association between patients with DM and outcome was therefore adjusted for age, sex, current smoking, body mass index and time period. CLTI, chronic limb-threatening ischaemia; DM, diabetes mellitus.

Nominal variables were described using frequencies and percentages. A Kolmogorov–Smirnov test was used to decide whether to use a parametric- or nonparametric statistical test for each variable. Nonparametric continuous variables were expressed as median and interquartile range (IQR), and evaluation of group differences between the data from 2001 and 2023 was performed using the Mann–Whitney *U* test. Parametric continuous variables were expressed as mean and standard deviation (SD), and evaluation of group differences was performed using independent sample *t* test. The Chi-square test was used to test differences in nominal variables between the data from 2001 and 2023, or between those with DM compared to those without DM in 2023. The associations between lipid-lowering treatment and time periods 2023 versus 2001 (fixed factors), and plasma cholesterol (dependent variable) were tested for confounding in a linear regression model. A minimum sufficient adjustment set for estimating the total effect of DM on major amputation/mortality at 1 year in patients with DM and CLTI was illustrated using a directed acyclic graph over potential interactions between variables (Figure 1). DM was the exposure and major amputation or mortality at 1 year are the outcomes. Confounders and mediators were identified, and confounding variables were entered as covariates in multivariable logistic regression model. Risks for major amputation or mortality at 1 year were expressed in odds ratios (OR) with 95% confidence intervals (CIs). All skewed exposure variables were log-transformed, and ORs were expressed per 1 SD increment. Missing values were left as missing, and no values were imputed. The significance level was set to *p* < 0.05. IBM Statistical Package for the Social Sciences (SPSS) (Chicago, IL, USA), version 29, was used for statistical analysis.

Incidence rates were calculated using the Poisson regression model, assuming Poisson distribution of events and expressed using patients per 100,000 person-years with 95% CIs. Background population data from the 19 communities in the catchment area of Skåne University Hospital in 2001 and 2023, retrieved from Statistics Sweden, were used to calculate the incidence rates.

## Results

### Comparison of incidence of hospitalised patients with CLTI between 2001 and 2023

Incidence rate of patients hospitalised for CLTI was 37.4 (95% CI, 33.3–41.6) persons per 100,000 person-years in 2001, compared to 22.8 (95% CI, 19.7–25.8) per 100,000 person-years in 2023.

### Comparison of risk factors, treatment and outcomes in hospitalised patients with CLTI between 2001 and 2023

Comparison of variables in hospitalised patients with CLTI between 2001 and 2023 is shown in Table 1. Compared to 2001, the patients in 2023 had a higher ankle-brachial index (ABI) (*p* < 0.001), lower diastolic BP (*p* < 0.001) and lower prevalences of both IHD (*p* = 0.046) and concomitant atherosclerotic diseases (*p* = 0.009). GFR was higher (*p* < 0.001), and plasma total and LDL cholesterol were lower (*p* < 0.001) in patients from 2023. The percentages of patients treated with full-dose oral anticoagulants (*p* < 0.001), angiotensin II receptor blockers or angiotensinogen converting enzyme inhibitors (*p* < 0.001), and lipid-lowering drugs (*p* < 0.001) were higher in 2023 compared to the group from 2001. No patients were newly diagnosed with DM during hospitalisation in 2023 compared to 4.2% in 2001. There were 136 (52.5%) patients with DM in 2001 compared to 112 (52.6%) in 2023 (*p* = 0.99). There were 48 (22.5%) patients with full anticoagulation due to atrial fibrillation in 2023 (not documented in 2001).

**Table 1.** Risk factors and outcomes for hospitalised patients with CLTI with and without DM in 2001 and 2023.

Study years	2001			2023		
Patients	All (*n* = 259)	DM (*n* = 136)	No DM (*n* = 123)	All (*n* = 213)	DM (*n* = 112)	No DM (*n* = 101)
Age (median ± IQR)	76 (68–82)	75 (67–82)	77 (70–82)	78 (71–82)	78 (70–82)	78 (71–83)
Female sex (%)	121 (46.7)	51 (37.5)	70 (56.9)	87 (40.8)	29 (25.9)	58 (57.4)
Body mass index (kg/m^2^) (median ± IQR)	24.2 (21.9–27.7) (*n* = 253)	25.0 (22.6–29.4 (*n* = 131)	23.6 (21.1–26.1) (*n* = 122)	25.0 (22.3–27.7) (*n* = 208)	26.0 (23.7–28.2) (*n* = 110)	23.6 (20.9–26.4) (*n* = 98)
Active smoking (%)	84/252 (33.3)	35/132 (26.5)	49 (40.8)	56/209 (26.8)	21/111 (18.9)	35/98 (35.7)
Foot ulcer (%)	206/256 (80.5)	113/134 (84.3)	93/122 (76.2)	175 (82.2)	107 (95.5)	68 (67.3)
Ankle-brachial index (median ± IQR)	0.32 (0.17–0.45) (*n* = 237)	0.32 (0.0–0.44) (*n* = 123)	0.33 (0.21–0.46) (*n* = 114)	0.52 (0.35–0.73) (*n* = 143)	0.62 (0.43–0.79) (*n* = 73)	0.44 (0.29–0.69)
Systolic BP (median ± IQR)	148 (130–160) (*n* = 254)	145 (130–160) (*n* = 133)	150 (130–160) (*n* = 121)	141 (129–159)	141 (127–158)	142 (131–159)
Diastolic BP (median ± IQR)	75 (70–80) (*n* = 249)	72 (70–80) (*n* = 131)	76 (70–82) (*n* = 118)	70 (63–76)	70 (63–77)	70 (63–76)
Hypertension (%)	236/257 (91.8)	128/135 (94.8)	108/122 (88.5)	199 (93.4)	109 (97.3)	90 (89.1)
Antihypertensive treatment (%)	211 (81.5)	116 (85.3)	95 (77.2)	179 (84.0)	101 (90.2)	78 (77.2)
Angiotensinogen converting enzyme inhibitors or Angiotensinogen receptor blockers (%)	97 (37.5)	58 (42.6)	39 (31.7)	128 (60.1)	72 (64.3)	56 (55.4)
Ischaemic heart disease (%)	123/258 (47.7)	73/135 (54.1)	50 (40.7)	82 (38.5)	49 (43.8)	33 (32.7)
Cerebrovascular accident (%)	70/258 (27.1)	40/135 (29.6)	30 (24.4)	44 (20.7)	25 (22.3)	19 (18.8)
Concomitant atherosclerotic disease	157/258 (60.9)	92/135 (68.1)	65 (52.8)	104 (48.8)	59 (52.7)	45 (44.6)
Plasma cholesterol (mmol/L; mean ± SD)	4.8 (1.2) (*n* = 253)	4.6 (1.2) (*n* = 133)	5.0 (1.2) (*n* = 120)	3.7 (1.1) (*n* = 171)	3.4 (1.0) (*n* = 98)	4.0 (1.0) (*n* = 73)
Plasma-LDL cholesterol (mmol/L) (median ± IQR)	2.8 (2.1–3.5) (*n* = 246)	2.6 (1.9–3.5) (*n* = 128)	3.0 (2.4–3.6) (*n* = 118)	1.9 (1.4–2.4) (*n* = 173)	1.7 (1.2–2.2) (*n* = 99)	2.1 (1.7–2.7) (*n* = 74)
Plasma-HDL cholesterol (mmol/L) (median ± IQR)	1.1 (0.8–1.4) (*n* = 251)	1.0 (0.8–1.2) (*n* = 132)	1.2 (0.9–1.5) (*n* = 119)	1.0 (0.8–1.4) (*n* = 173)	0.9 (0.7–1.2) (*n* = 99)	1.4 (1.0–1.6) (*n* = 74)
Plasma triglycerides (mmol/L) (median ± IQR)	1.4 (1.1–2.1) (*n* = 252)	1.6 (1.0–2.4) (*n* = 132)	1.4 (1.1–1.8) (*n* = 120)	1.4 (1.0–2.0) (*n* = 173)	1.6 (1.1–2.2) (*n* = 100)	1.2 (0.9–1.8) (*n* = 73)
Platelet aggregation inhibitors (%)	180 (69.5)	94 (69.1)	86 (69.9)	147 (69.0)	65 (58.0)	82 (81.2)
Full-dose oral anticoagulants (%)	32 (12.4)	18 (13.2)	14 (11.4)	83 (39.0)	49 (43.8)	34 (33.7)
On lipid-lowering treatment (%)	61 (23.6)	36 (26.5)	25 (20.3)	182 (85.4)	95 (84.8)	87 (86.1)
Anaemia (%)	150 (57.9)	89 (65.4)	61 (49.6)	128 (60.1)	75 (67.0)	53 (52.5)
Glomerular filtration rate (mean ± SD)	60 (27) (*n* = 258)	57 (28) (*n* = 135)	63 (25)	73 (29)	68 (33)	78 (22)
Vascular surgery, any (%)	192 (74.1)	104 (76.5)	88 (71.5)	166 (77.9)	85 (75.9)	81 (80.2)
Endovascular therapy (%)	128 (49.4)	71 (52.2)	57 (46.3)	129 (60.6)	78 (69.6)	51 (50.5)
Open vascular surgery (including hybrid vascular surgery) (%)	64 (24.7)	33 (24.3)	31 (25.2)	37 (17.4)	7 (6.2)	30 (29.7)
Major amputation at 1 year (%)	47/250 (18.8)	29/134 (21.6)	18/116 (15.5)	39 (18.3)	27 (24.1)	12 (11.9)
Mortality at 1 year (%)	62 (23.9)	36 (26.5)	26 (21.1)	37 (17.4)	24 (21.4)	13 (12.9)
Major amputation/mortality at 1 year (%)	92/255 (36.1)	55 (40.4)	37/119 (31.1)	62 (29.1)	40 (35.7)	22 (21.8)

BP, blood pressure; CLTI, chronic limb-threatening ischaemia; DM, diabetes mellitus; IQR, interquartile range.

When entering plasma cholesterol as a dependent variable and time period (2023 vs 2001) and lipid-lowering treatment as fixed factors in a linear univariate regression analysis, plasma cholesterol values remained lower in 2023 compared to 2001 (*p* = 0.026).

### The association between DM and outcome in patients with CLTI

When entering age, gender, time period (2023 vs 2001), BMI, current smoking and DM as covariates, and major amputation or mortality at 1 year as a dependent variable in a logistic regression model, age (OR, 1.50/1 SD increase, 95% CI, 1.18–1.90) and DM (OR, 1.72, 95% CI, 1.11–2.66) were independently associated with increased odds of major amputation/mortality at 1 year. When entering the same variables as covariates, and mortality at 1 year as dependent variable, age (OR, 2.38/1 SD increase, 95% CI, 1.69–3.37) and DM (OR, 1.70, 95% CI, 1.02–2.83) were independently associated with increased odds of mortality at 1 year, whereas time period (2023 vs 2001; OR, 0.62, 95% CI, 0.38–0.99) was associated with decreased odds of mortality.

### Comparison of risk factors, treatment and outcomes in hospitalised patients with CLTI and DM between 2001 and 2023

Comparison of variables in hospitalised patients with CLTI and DM between 2001 and 2023 is shown in Table 1. Similar differences were seen as in the whole study population. Endovascular therapy (*p* = 0.005) was performed more often in 2023, whereas open vascular surgery (*p* < 0.001) was performed less often.

### Comparison of risk factors and outcomes in hospitalised patients with CLTI with DM compared to those without DM in 2023

Anaemia was present at admission in 67.0% (75/112) and 52.5% (53/101) of patients with CLTI with and without DM, respectively (*p* = 0.031; Table 1). Endovascular therapy was performed more often in those with DM compared to those without DM (*p* = 0.004). Major amputation at 1 year (*p* = 0.021) and major amputation or mortality at 1 year (*p* = 0.025) occurred more often in those with DM compared to those without DM, whereas there was no difference between the groups in mortality at 1 year (*p* = 0.10).

### Comparison of adherence to the GVG in patients with CLTI with and without DM in 2023

Toe pressure measurements (*p* = 0.003) were more often performed in patients with DM (Table 2), whereas patients with DM were less often offered smoking cessation interventions (*p* = 0.033), antiplatelet therapy (*p* = 0.008) and questioned about physical activity (*p* < 0.001). WIfI classification in patients with foot ulcers was documented in 6.9% of the patients, without any difference in those with and without DM. Among obese patients, 12.9% were given advice against obesity, and diet advice was given to 10.3% of the patients, without group differences.

**Table 2.** Adherence to global vascular guidelines on risk factors at hospital admission or discharge for CLTI with or without DM in 2023.

Variables	All (*n* = 213)	DM (*n* = 112)	No DM (*n* = 101)	*p*-Value
Ankle pressure measured (%)	160 (75.1)	83 (74.1)	77 (76.2)	0.72
Ankle-brachial index measured (%)	159 (74.6)	83 (74.1)	76 (75.2)	0.85
Toe pressure or toe-brachial index measured (%)	163 (76.5)	95 (84.8)	68 (67.3)	0.003
Ankle or toe-brachial index measured (%)	201 (94.4)	106 (94.6)	95 (94.1)	0.85
Question about smoking (%)	205 (96.2)	107 (95.5)	98 (97.0)	0.72
Offered any smoking cessation intervention in smokers^a^ (%)	43/67 (64.2)	12/25 (48.0)	31/42 (73.8)	0.033
Questioned about physical activity (%)	128 (60.1)	43 (38.4)	85 (84.2)	<0.001
Advice on diet (less carbohydrates and less saturated fats, more fruit and vegetables) (%)	22 (10.3)	11 (9.8)	11 (10.9)	0.80
Advice against obesity if obese (BMI ⩾ 30 kg/m^2^)^a^ (%)	4/31 (12.9)	2/19 (10.5)	2/12 (16.7)	0.63
Platelet aggregation inhibitor (at admission or discharge) (%)	157 (73.7)	74 (66.1)	83 (82.2)	0.008
Platelet aggregation inhibitor and low-dose rivaroxaban (at admission or discharge) (%)	67 (31.5)	26 (23.2)	41 (40.6)	0.006
Full-dose oral anticoagulants (%)^b^	83 (39.0)	49 (43.8)	34 (33.7)	0.13
Any anticoagulant therapy (%)^b^	150 (70.4)	75 (67.0)	75 (74.3)	0.24
Moderate or high-intensity statins at discharge (%)	144 (67.6)	72 (64.3)	72 (71.3)	0.28
Lipid-lowering treatment at discharge (%)	157 (73.7)	85 (75.9)	72 (71.3)	0.45
Control of systolic BP during hospitalisation (%)	213 (100.0)	112 (100.0)	101 (100.0)	–
Control of diastolic BP during hospitalisation (%)	213 (100.0)	112 (100.0)	101 (100.0)	–
Metformin in type 2 DM (%)^a^	52/108 (48.1)	52/108 (48.1)	–	–
WIfI classification, all patients (%)	13 (6.1)	5/107 (4.5)	8 (7.9)	0.29
WIfI classification if foot ulcer (%)	12/175^a^ (6.9)	5/107 (4.5)	7/68 (10.3)	0.15
Assessment of neuropathy (%)	169 (79.3)	89 (79.5)	80 (79.2)	0.96

a Number of patients receiving the GVG recommendation/total number of patients encountered with the specific condition or diagnosis. Three patients had type 1 DM and one patient had diet treated type 2 DM.

b Data added to show the total number of patients with anticoagulant therapy.

BMI, body mass index; BP, blood pressure; CLTI, chronic limb-threatening ischaemia; DM, diabetes mellitus; WIfI, Wound, Ischaemia and foot Infection.

## Discussion

It appears that the incidence of CLTI is declining in the southernmost part of Sweden. Considering the general decrease in atherosclerotic diseases in Sweden,^10^ it seems likely that the observed decline in incidence of hospitalisations for CLTI could be true and related to lower rates of smoking^11^ and better secondary medical preventive therapy against atherosclerosis.^12^ However, the decrease in incidence could also be dependent on organisational changes in health care, such as a lower number of available beds at the Department of Vascular Diseases in 2023. The corona virus disease (COVID-19) pandemic was declared no longer a public health emergency on 5 May 2023, by WHO.^13^ During the pandemic the whole healthcare system was affected, resources needed to be shifted, leading to cancelled appointments and surgeries, and the patients’ care-seeking behaviours changed.^14^ This healthcare crisis during the pandemic might probably have remained to some extent into the post-pandemic period, which could have lowered the incidence of hospitalised patients with CLTI. Furthermore, there is a shortage of nurses in Swedish health care, adding to the decrease in access to health care for patients with CLTI, particularly for those in need of hospitalisation,^15^ which also might have contributed to the lower incidence.

The present study indeed showed an improved medical care of CLTI in 2023 compared to 2001. There were no newly detected patients with previously undiagnosed DM during hospitalisation in 2023. Better adherence to national guidelines regarding diabetes care^16^ might have led to earlier diagnosis of DM and may also explain why fewer patients with DM are nowadays diagnosed with CLTI. The lower plasma cholesterol levels in 2023 are most probably related to an increased use of lipid-lowering treatments. Nevertheless, after adjusting for lipid-lowering treatment and time period 2023 versus 2001, plasma cholesterol remained significantly lower in 2023. This indicates that other factors, perhaps healthier diets or improved physical activity, contributed to the lower plasma cholesterol values in 2023. The significantly higher rate of lipid-lowering therapy could have reduced the concomitant burden of atherosclerotic diseases, including renal artery disease, resulting in a better renal function in the latter period. These factors likely contribute to the reduced odds of mortality in the latter period.^17,18^

Endovascular therapy is a minimal-invasive therapy which is performed under local anaesthesia, and logistics, equipment and skills have developed greatly during the last decades. It was therefore of no surprise that endovascular therapy was more often performed during the latter study period, particularly in patients with CLTI and DM. Patients with DM may be more fragile and have more distal occlusive arterial lesions making them more suitable for endovascular therapy.^19^ The mode of therapy was considered as a mediator, and not a confounder, and was therefore not adjusted for in the multivariable analyses.

The present study identified a low adherence to current recommendations according to the GVG^1^ regarding WIfI classification of hospitalised patients with foot ulcer, diet advice and advice against obesity. There was also an inadequate rate of offered smoking cessation interventions. There are very few reports about adherence to guideline directed therapies in patients with CLTI. However, in a recent study on adherence to guideline recommended medical therapies in type 2 DM patients with CLTI, a low application of guideline directed therapies was noted.^20^ In view of their study, the present study showed a somewhat better adherence to guideline directed medical therapies in patients with and without DM. The low percentage of WIfI classification of foot ulcers, 6.9%, suggests that this system is not yet an established routine, however, neither among vascular surgeons nor endocrinologists.

A diet resulting in excessive levels of LDL cholesterol and glucose is another risk factor adversely affecting the atherosclerotic process in the arteries,^21,22^ but only 10.3% of the patients were given dietary advice. An observation worth mentioning, however, is that many patients in the study had problems with involuntary weight loss and were given advice on how to increase the intake of foods with high energy levels. From this observation, it could be assumed that it has been more important to focus on these patients’ energy intake rather than how healthy their diets are. Aside from that, there is room for improvement regarding dietary advice to patients with CLTI.

Obesity is another risk factor contributing to the development of atherosclerosis,^1^ but only 12.9% of obese patients with obesity were offered advice against the condition. The reason behind this low percentage could be the sensitivity of the subject or that obesity was considered as not important enough to address in the patient’s current situation. A better way to implement the importance of obesity could be to make registration of BMI mandatory in the patient records and to educate all personnel on risk factor management.

Nearly all patients, 96.2%, were asked about smoking but only 64.2% of the active smokers were offered any smoking cessation intervention, a percentage that should be improved. A lower proportion of patients with DM was offered any smoking cessation intervention, which is alarming, since smoking and DM is an ominous combination for atherosclerotic disease progression.^23^

The high adherence in performing ankle- or toe-brachial index measurements, 94.4%, must be considered acceptable. In a few patients, there was simply no possibility of registration and documentation of distal arterial pressures due to ulcers, amputations or unreliably very high arterial pressures due to noncompressible calcified arteries.

The overall rates of secondary preventive medical therapy such as antiplatelet therapy alone or combined with low-dose rivaroxaban (the regime used in the Voyager PAD study) and moderate or high-intensity statin treatment were reasonably good. The lower rates of prescription of antiplatelet therapy with or without low-dose rivaroxaban among patients with DM was probably related to the higher rates of patients on full-dose anticoagulation in this group.

Another recommendation from the GVG from 2019 was to use metformin as the primary glucose lowering agent.^1^ As newer anti-diabetic drugs such as sodium-glucose cotransporter-2 inhibitors and glucagon-like peptide-1 receptor agonists lower the incidence of major adverse cardiovascular events in diabetes,^24,25^ they are now recommended instead of metformin as first-line therapy for type 2 DM in patients with atherosclerotic disease^24,25^ which might explain the relatively low number of patients on metformin. A prospective study specific on patients with DM and CLTI showed that lower Klotho protein and higher fibroblast growth factor 23 levels measured at baseline were independently associated with major adverse cardiovascular and limb-related events at 1 year, suggesting a modulating role for these proteins in the atherosclerotic pathways and possible targets for future anti-atherosclerotic drugs.^26^

Questions about physical activity were asked in 80.5% of the cases, which is considered acceptable. It was not addressed whether the patients should improve their activity level if considered low according to the recommendations from WHO,^6^ however, and few recommendations on physical activity were given. This is another field in which there is room for future improvement.

The evidence regarding alcohol consumption is scarce and the recommendations in the GVG^1^ are therefore few, but evidence for improved recovery after surgery when refraining from alcohol prior to surgery does exist.^27,28^ Therefore, a recommendation on alcohol consumption should be included in the next updated GVG on the management of CLTI.

Recommendations regarding treatment of anaemia in patients with CLTI are not included in the GVG.^1^ There is however some evidence for worse clinical outcomes and higher mortality rate in patients with CLTI and concomitant anaemia,^29^ which indicates that the high percentage of patients registered with anaemia, 67.0% in those with DM and 52.5% in those without DM, is another possible area of improvement.

The present retrospective study has elements of selection and information bias. Even though some confounding variables were adjusted for, there could be others not identified or accounted for such as ethnicity and socio-economic inequities.^30^ No sample size calculation was performed to estimate assumed differences in outcomes between the years 2023 and 2001, since this is an explorative study. A strength of the present study is that the two sets of collected data, in 2001 and 2023, are more than two decades apart. This enables the possibility to reflect on changes in clinical practice regarding hospitalised patients with CLTI. Another strength is the use of direct acyclic graph as a basis for adjustment of confounders.

## Conclusion

During the two last decades, the incidence of hospitalisation for CLTI appears to have been reduced, and medical care of patients with CLTI has improved significantly leading to an improved prognosis. Physicians have not yet adopted the WIfI classification in patients with CLTI, and better routines for diet advice and smoking cessation interventions need to be implemented. There is still room for large improvements of secondary preventive care in patients with CLTI, particularly in those with DM.

## Declarations

**Ethics approval and consent to participate:** This study was approved by the Swedish Ethical Review Authority (Dnr 2024-01390-01). Informed consent was waived by the Swedish Ethical Review Authority (Dnr 2024-01390-01).

**Consent for publication:** Not applicable.

**Availability of data and materials:** The raw data supporting the conclusions of this article will be made available by the authors, without undue reservation.
